# The School Clinic

**Published:** 1911-11-11

**Authors:** 


### THE SCHOOL CLINIC.

### III.—The Parents' Point of View.

To the average parent of a school-going child the question of medical supervision presents itself in rather a different light from that which it shows to the general practitioner. Moreover, it presents a different aspect to the well-to-do parent from that in which the poor man is likely to regard it. The former, who has the means and the opportunities to have his child examined by his private medical attendant, usually regards inspection by the school doctor as a superfluity which may well be dispensed with. Any recommendation for treatment in such a case is equally likely to be regarded as an impertinence on the part of the school doctor. The parent argues that the family doctor knows much more about the child's constitution and idiosyncrasy than the school doctor possibly can, and in any case, even where the recommendation for treatment is accepted and acted upon, the child will be taken to the family practitioner, and certainly not to a clinic.

The poor parent, on the other hand, looks at things in a totally different light. Excluding the very small percentage of obstinate parents who object to medical inspection on the same grounds as the Irishman objected to Governments—a percentage which cannot, in the present state of the law, be reached by the medical inspectors, but can only be coped with by the exercise of great tact, good temper, and persuasion on the part of both doctor and nurse—the large majority of poor parents undoubtedly welcome inspection and supervision by the school doctor. The difficulty they have is entirely with regard to treatment. Such a parent gets the stereotyped advice, "Your boy or girl is suffering from a diseased condition of the nose or throat which, if not seen to, will undoubtedly interfere with his prospects at school; you must take him somewhere for treatment." The natural query is, Where? It is easy enough to answer, "To your own doctor." Mostly such poor parents have no regular family attendant; they belong to the huge class of club patients or to the still more huge section of those who crowd our out-patient departments. They cannot very well afford to take the child to a local doctor, who in the majority of cases does not find it worth his while to do an operation, even so minor a one as scraping for adenoids, for the price he charges for his bottle of mixture and advice. They cannot afford to pay him adequately for his skill and time. On the other hand, they know, by personal experience, the hardship entailed on them by taking the child to an out-patient department. Apart from the prolonged waiting, such a course necessitates the attendance of the mother or father; at any rate of an adult relative, who will have to give up at least a morning or afternoon, and consequently the amount of wages which might have been earned during that period. A small matter to the school doctor who draws his salary from the rates, but by no means an unimportant consideration to the average poor parent! It is to such parents that the school clinic forcibly appeals. Even when the treatment is not gratuitous, it is at all events graduated, or should be graduated, to suit the capacities of the case; there is less waiting; there is a certain amount of necessary supervision; and there is a reasonable prospect that the operation, or the treatment, whatever it may be, will be done as efficiently as at a hospital or by a private practitioner.

With the ethics of the school clinic the average parent has nothing whatever to do. Whether it is staffed by expert consultants, by school doctors, or by local practitioners, does not matter to him. The very fact that it is authorised, that it is close at hand, that it affords a means of following the recommendation given by the medical inspector, is sufficient for him. Probably he would have greater confidence in hospital treatment, for notwithstanding the fact that the poor man still has an aversion to hospitals—a fact with which every practitioner in South London, for instance, is perfectly well acquainted—he has a sneaking suspicion that the doctor at the hospital is a much more clever person than the doctor in his own neighbourhood. But his great objection to hospital treatment is the attendant difficulty of securing it without having to pay what to him is a comparatively extravagant price. This is a point which the hospital worker apparently does not yet grasp. He does not realise that, although the treatment at out-patients is given freely and gratuitously, it necessarily exacts from the parent a charge which in most cases is measured in time that in the case of a worker is easily translated into money. Under the American system, which may possibly be adduced as a substitute for school clinics, the school nurse is empowered to take the child to the hospital. This is certainly an advantage, but it means the increase of the nursing staff, and at the same time a division of responsibility to which the parent may legitimately object. What the parent wants is the means to carry out the school doctor's recommendation with the minimum loss of time and at the minimum of expense to himself. And the only way of securing this is by the establishment of a school clinic in each district to which physically defective children may be taken. Such a treatment centre is imperatively required in cases which need more than one attendance for their cure. The cases which require operation or in-patient treatment must necessarily go to the hospital, but then there is no choice in the matter, and the parent must adapt himself to circumstances. Nor does he as a general rule decline to do so. It is the universal experience that parents are only too willing to take their children for hospital treatment in serious conditions; it is the minor defect, whose results in the long run are so pernicious, that is neglected. And it is just this kind of case which demands the establishment of the school clinic.

